# Myalgic Encephalomyelitis/Chronic Fatigue Syndrome as a Hyper-Regulated Immune System Driven by an Interplay Between Regulatory T Cells and Chronic Human Herpesvirus Infections

**DOI:** 10.3389/fimmu.2019.02684

**Published:** 2019-11-21

**Authors:** Nuno Sepúlveda, Jorge Carneiro, Eliana Lacerda, Luis Nacul

**Affiliations:** ^1^Department of Infection Biology, Faculty of Infectious and Tropical Diseases, London School of Hygiene & Tropical Medicine, London, United Kingdom; ^2^Centre of Statistics and Its Applications, University of Lisbon, Lisbon, Portugal; ^3^Quantitative Organism Biology Group, Gulbenkian Institute of Science, Oeiras, Portugal; ^4^Department of Clinical Research, Faculty of Infectious and Tropical Diseases, London School of Hygiene & Tropical Medicine, London, United Kingdom

**Keywords:** viral infection, autoimmunity, immunological theory, immunological tolerance and regulation, hypothesis, mathematical modeling, disease pathology

## Abstract

Autoimmunity and chronic viral infections are recurrent clinical observations in Myalgic Encephalomyelitis/Chronic Fatigue Syndrome (ME/CFS), a complex disease with an unknown cause. Given these observations, the regulatory CD4+ T cells (Tregs) show promise to be good candidates for the underlying pathology due to their capacity to suppress the immune responses against both self and microbial antigens. Here, we discussed the overlooked role of these cells in the chronicity of Human Herpes Virus 6 (HHV6), Herpes Simplex 1 (HSV1), and Epstein–Barr virus (EBV), as often reported as triggers of ME/CFS. Using simulations of the cross-regulation model for the dynamics of Tregs, we illustrated that mild infections might lead to a chronically activated immune responses under control of Tregs if the responding clone has a high autoimmune potential. Such infections promote persistent inflammation and possibly fatigue. We then hypothesized that ME/CFS is a condition characterized by a predominance of this type of infections under control of Tregs. In contrast, healthy individuals are hypothesized to trigger immune responses of a virus-specific clone with a low autoimmune potential. According to this hypothesis, simple model simulations of the CD4+ T-cell repertoire could reproduce the increased density and percentages of Tregs observed in patients suffering from the disease, when compared to healthy controls. A deeper analysis of Tregs in the pathogenesis of ME/CFS will help to assess the validity of this hypothesis.

## Introduction

ME/CFS is a debilitating illness of unknown cause characterized by a persistent fatigue, post-exertion malaise, unrestful sleep, frequent infections, and other non-specific symptoms (1, 2). Most of the patients trigger the disease during adulthood after a viral infection (3). Human herpes 6 virus (HHV6), Epstein–Barr virus (EBV), and Herpes simplex virus 1 (HSV1) are just a few examples of possible infection triggers reported for the disease (4). As a possible consequence of these infections, patients often show an activated immune system with the presence of a high quantity of autoantibodies. Given these observations, some authors hypothesized an autoimmune origin for the disease (5, 6). If true, an impairment of key immune-cell populations controlling autoimmunity, such as the regulatory T CD4+ cells (Tregs), should be implicated in the pathogenesis of ME/CFS.

Tregs represent a small subset of the CD4+ T cells that are able to suppress the effector activity of conventional CD4+ T cells (Teffs) and other immune cells. These cells are molecularly identified by the activation marker CD25 and the transcription factor FOXP3 (7, 8). The constitutive expression of the *foxp3* gene in CD4+ T cells after thymic T-cell development is the hallmark of the natural Tregs (9). In turn, a subset of CD4+ T cells can express the *foxp3* gene in the periphery upon antigen stimulation. These cells are called induced or adaptable Tregs (9). Both types of Tregs are thought to recognize tissue antigens mainly, which ensures the regulation of potentially damaging responses against the body. This hypothesis comes from the observation that the deletion of Tregs or the suppression of their regulatory activity leads to severe and generalized autoimmune responses in inbred mice and humans (10–12). In addition, a reduction of these cells in the periphery is in the origin of naturally occurring type I diabetes in the NOD mouse strain (13).

Until now, the role of Tregs on the pathogenesis of ME/CFS has been simply assessed by comparing the respective cell counts between patients and healthy controls. With the exception of a single study (14), the percentage of Tregs tends to be increased in patients when compared to healthy controls (15–17). Similar tendency was found for the transforming growth factor beta (TGFβ), the Treg-associated suppression cytokine (18, 19). These clinical observations were considered a paradox under the postulated autoimmune origin for ME/CFS (5). However, they prompted us to consider an alternative hypothesis for the pathogenesis of ME/CFS according to which Tregs are elevated resulting from chronic infections that are cross-reactive with self-antigens.

The present paper aims then to present different T-cell and viral dynamics consistent with this hypothesis using the cross-regulation model for the immune-physiology of Tregs (20–22). With this purpose, we first introduce the basic immunological theory suggested by this model. We then extend this theory for the role of Tregs in the presence of HHV6, EBV, and HSV1 infections, which helps to discuss their impact on Tregs and on the pathogenesis of ME/CFS.

## The Cross-Regulation Model for CD4+ T-Cell Dynamics and Its Extension for Chronic Viral Infections

**Figure 1:**
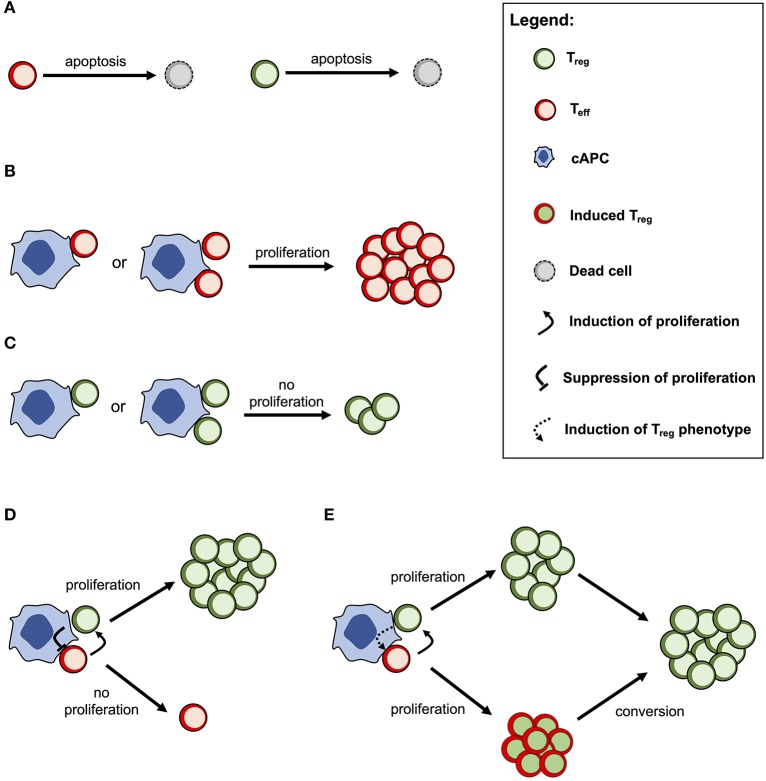
Conceptual scheme of the cross-regulation model under the assumption of cAPC with two conjugation sites. **(A)** Tregs and Teffs will eventually die by cellular apoptosis if they are not conjugated and activated by their cAPC. **(B)** Teffs are able to proliferate when conjugated with their cAPCs occupied by other Teffs. **(C)** Tregs are not able to proliferate when conjugated with a cAPC occupied by other Tregs. **(D)** When Tregs and Teffs occupy different conjugation sites in the same cAPC, the former proliferates by receiving a signal (or a growth factor) from the latter. At the same time, Tregs send a signal that suppresses the proliferation of Teffs. **(E)** When Tregs and Teffs occupy different conjugation sites in the same cAPC, both cell populations are able to proliferate, but the latter will convert to a Treg phenotype after some time. This alternative mechanism is mathematically equivalent to the one shown in **(D)** but the proliferation rate of Tregs is expected to be higher than the one of Teffs.

The cross-regulation model describes the dynamics of Tregs and effector T cells (Teffs) and their mutual interaction dependent on multicellular conjugates with cognate antigen-presenting cells (cAPCs) (Figure 1) (20, 23). Conjugation and deconjugation with cAPC are assumed to be the basic cellular process by which Tregs and Teffs become activated and then proliferate; otherwise, they would die by apoptosis with a given rate (Figure 1A). The model assumes that Teffs can only proliferate following productive conjugations with their cAPCs in absence of Treg co-conjugation (Figure 1B). In contrast, Tregs can only proliferate when co-conjugated with Teffs on the same cAPC (Figure 1C). In that case, Treg proliferation occurs upon receiving growth signals or factors provided by Teffs. At the same time, Tregs are assumed to send a molecular signal (e.g., via TGFβ) that inhibits the proliferation of Teffs (Figure 1D). It is worth noting that this mechanism is mathematically equivalent to a related one where some Teffs are induced to become Tregs (Figure 1E). If these two mechanisms are in place, then the proliferation rate of Tregs should be increased in relation to the one of Teffs. A detailed discussion supporting these model assumptions can be found elsewhere (20). Mathematically speaking, the model used in this article was deeply simplified, but still captures the complex non-linear effects of Treg immunophysiology and its conjugation with cAPC. A detailed description of this simplified model is provided in Supplementary File.

For simplicity, the CD4+ T-cell repertoire of a given individual is divided into multiple independent clones containing both Tregs and Teffs. Each clone is associated with a specific set of cAPC with a given abundance in the periphery and the respective dynamics is described by the cross-regulation model. Independence between clones in the repertoire simplifies the analysis as one only needs to interpret all possible dynamics of a single clone associated with a given set of cAPC. This implies that Tregs and Teffs from the same clone cross-regulate each other, but the same cells from different clones do not; a model assuming a cross-regulation between distinct clones can be found elsewhere (21).

In this discussion article, we also consider an extension of the basic cross-regulation model to the scenario where individuals are infected by HHV6, HSV1, and EBV at some point of their lifetime. The detailed description of the respective mathematical model is presented in Supplementary File. The main purpose of this extended model is to provide a quantitative framework where the role of Tregs on the pathogenesis of ME/CFS can be discussed.

## Immunological Theory Based on the Cross-Regulation Model

A simple immunological theory can be constructed using a steady-state analysis of the basic cross-regulation model (20). This theory helps to understand how autoimmunity, microbial immunity, and ME/CFS can be reconciled. In this theory, the peripheral CD4+ T-cell repertoire is assumed to be composed of independent clones that allow the determination of the steady states of a generic T-cell clone in isolation as function of the abundance of the respective cAPC in the periphery, while considering the remaining model parameters fixed at their reference value. For this analysis, we introduce the term “autoimmune potential” of a particular clone as the abundance of the associated cAPC in the periphery.

**Figure 2:**
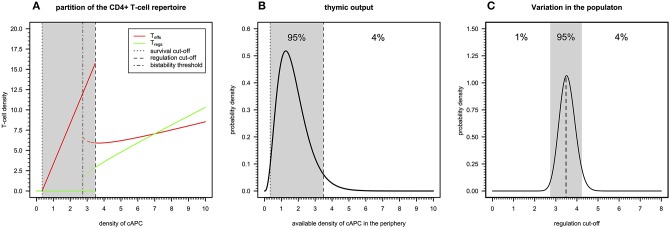
Basic immunological theory based on the cross-regulation model. **(A)** The steady-states for the density of Teffs and Tregs belonging to a given clone as a function of the density of a given cAPC where dotted, dotted–dashed, and dashed vertical cutoffs refer to the limits on cAPC density for T-cell survival, bi-stability, and regulation by Tregs. The shaded area represents the cAPC densities that can only maintain Teffs in equilibrium under initial conditions of 0.90 and 0.10 for Teffs and Tregs, respectively. The bi-stability cutoff represents the minimum cAPC density upon which system reaches a bi-stable regime. That is, depending on the initial conditions for Tregs and Teffs, the system would reach either a steady state with Teffs (solid red line) or a steady state with co-existence of Teffs and Tregs (dashed red and green lines, respectively). For the generation of this plot, the cross-regulation model was simulated for 25,000 units of time and for each value of cAPC density with the remaining parameters fixed at the respective reference values (Table S1). **(B)** Example of a possible distribution of clones generated by a thymic output of a healthy individual, where the shaded area represents the cAPC densities associated with clones with an intermediate autoimmune potential. The limits of the shaded area refer to the T-cell survival and regulation cutoff values shown in **(A)**. For illustrative purposes, the distribution of clones follows a Gamma random variable whose shape and rate parameters (θ_1_ = 3.84 and θ_2_ = 2.25, respectively) were determined by equating the hypothetical 1% and 96% quantiles with the survival and regulation cutoff values shown in **(A)**. **(C)** Possible natural variation of the regulation cutoff in a given population assuming that the shaded area represents the regulation cutoff values associated with putative healthy individuals of the population. Again, for illustrative purposes, the distribution was illustrated with a Gamma probability distribution whose shape and rate parameters (θ_1_ = 89.24 and θ_2_ = 25.13, respectively) were determined by equating the 1% and 96% quantiles to the arbitrary values 0.15 and 0.25, respectively.

There are three qualitatively distinct steady states for the dynamics of a clone depending on its autoimmune potential (Figure 2A). On one extreme, there is a steady state where this clone undergoes extinction because its autoimmune potential is below a critical persistence (or survival) cutoff. This clone is expected to react to either microbial antigens or very rare tissue antigens before undergoing extinction (20). In the context of viruses with a strong CD4+ T-cell tropism, clonal extinction might be beneficial for the host as it exhausts the cellular source used for viral replication. On the other extreme, there is a steady state in which both Tregs and Teffs can be maintained together in the same clone, because the autoimmunity potential of the clone is above a critical regulation cutoff. This clone is predicted to recognize and react to ubiquitous tissue antigens whose immune responses against them are under control of Tregs in absence of infection. However, if a microbial antigen has by chance a high molecular mimicry with these self-antigens, then the respective microbe might become chronic due to a permanent suppression of the underlying immune response promoted by Tregs. Such situation is expected to contribute to a chronic inflammation and fatigue often associated with ME/CFS. The last steady state emerges between these two extreme cases and results from the dynamics of a clone where only Teffs can be maintained at equilibrium. In theory, this clone has an intermediate autoimmune potential and, thus, it should react to less abundant self-antigens and/or infections. By hypothesis, putative autoimmunity responses induced by the Teffs of this clone are under check by a bystander suppression that the model does not account for.

The peripheral CD4+ T-cell repertoire of a given individual should be then partitioned into three types of clones according to the above steady states. In theory, T-cell development in the thymus should impose qualitative restrictions on the fraction of clones associated with each of the steady states. On one hand, clones that just respond to very rare antigens are likely to not encounter their cognate antigens during T-cell development and, therefore, they would be purged from the repertoire due to lack of enough stimuli. On the other hand, developing clones recognizing highly abundant antigens with high affinity should be removed from the T-cell repertoire as they might trigger deleterious autoimmune responses when reaching the periphery. Then, a “healthy” thymic output should consist of a few clones that responding to rare or highly abundant antigens and a large number of clones that recognize antigens with intermediate abundance in the periphery. The “true” distribution of thymic output is of course unknown, but it might resemble the one illustrated in Figure 2B.

The above interpretation for the peripheral T-cell repertoire of a single individual can be then extended to a whole population of individuals. In this regard, polymorphisms in genes affecting physiology of CD4+ T cells (e.g., CTLA-4, CD25, or IL2), might contribute to individual variation on different model parameters affecting T-cell immune-physiology (e.g., T-cell proliferation and death rates). As a consequence, this variation is then translated into a heterogeneity of the regulation cutoff values present in the population. In this line of thought, the genetic predisposition of some individuals to autoimmunity or weak microbial immunity can be then re-interpreted in terms of variations in the regulation cutoff. Considering the thymic output shown in Figure 2B as the reference, individuals with genetic factors implying too-low regulation cutoffs are expected to be able to maintain too many Tregs in the peripheral repertoire. The excess of these cells is likely to suppress beneficial immunity against microbes. On the other hand, individuals with genetic factors predisposing to too-high regulation cutoffs are expected to show too many unregulated autoreactive clones in the peripheral repertoire. This situation contributes to the spontaneous onset of an autoimmune disease. Unfortunately, mathematical modeling cannot *per se* resolve the question of what can be considered too low or too high for the regulation cutoff. On top of that, the regulation cutoff can only be determined numerically given a set of model parameters, which brings additional difficulties in studying how variation in model parameters affects this quantity. However, for simplicity of the argument, one can easily conceive the existence of two disease-causing thresholds in a hypothetical distribution of regulation cutoffs in the population, as illustrated in Figure 2C. The exact proportion of healthy individuals, autoimmune patients, and patients with weak antimicrobial immunity is unknown, but one can speculate that infectious diseases should, in principle, exert a stronger genetic selection in the human genome than autoimmune diseases do. If so, individuals with a high predisposition for an autoimmune disease are expected to be slightly more frequent in the population than the ones affected by infections.

Given this basic immunological theory based on the cross-regulation model, it is reasonable to question where patients suffering from ME/CFS are placed in the distribution of the regulation cutoff. On the one hand, patients of this disease often show clinical symptoms similar to the ones presented by patients with autoimmune diseases, such as rheumatoid arthritis and multiple sclerosis (39, 40). On the other hand, patients suffering from ME/CFS do not develop the disease spontaneously as expected from a pathology with a strong genetic component. A classic example of a spontaneous autoimmune disease has been observed in NOD mice whose genetic mutations on CTLA-4 and CD25 genes elicit type 1 diabetes (41). Instead, patients suffering from ME/CFS report an infection at their disease onset. Given this observation, one can hypothesize that these patients might be healthy individuals who, by chance, were infected with a microorganism with a strong molecular mimicry to a human protein. This hypothesis will be discussed below in the context of the chronic infections by HHV6, HSV1, and EBV.

## Hypothetical Impact of Common Herpesviruses on CD4+ T-Cell Dynamics and Its Relationship With ME/CFS

Infections by EBV, HSV1, and HHV6 are highly frequent and chronic rather than transient in the human populations (24). Primary infection often occurs during childhood, after which these viruses enter a chronic phase with some reactivation over time under stress conditions, as observed in astronauts (42, 43). However, viruses are often found active in patients suffering from ME/CFS (4). Consistently with the theory based on the immunological homunculus (44, 45), these viruses are able to produce proteins that could mimick dominant self-antigens (Table 1) (30–32, 34). Antigen mimicry between these viral antigens and dominant self antigens has then the potential of initiating and perpetuating an autoimmune disease if the underlying immune response is not properly regulated. On the other hand, regulation of the immune response does not promote clearance of these viruses upon infection. In the context of the cross-regulation model, the link between viral molecular mimicry and self-reactivity is understood as function of the total abundance of the self-antigen and the respective viral antigen available for T-cell recognition in the body.

**Table 1:** Basic information about HHV6, HSV1, and EBV and their putative impact on the parameters of the cross-regulation model.

**Virus**	**HHV6**	**HSV1**	**EBV**
Age of primary infection (in years)	0.5–2 (24)	Childhood (25)	~20 (24)
Cell association during primary infection	CD4+ T cells (24)	Skin epithelial cells (24)	Oropharyngeal epithelium, B cells (24)
Cell association during latency	Macrophages (24)	Neurons (25)	B cells (26)
Virus-specific Tregs	Yes (27)	Yes (28)	Yes (29)
Possible antigen mimicry	Myelin basic protein (30)	Acetylcholine receptor (31), α-synuclein (32)	HNRNL (33), septin-9, mitochondrial protein DLST (34)
Possible mechanism of immune evasion	Inhibition of proliferation in CD4+ T cells (35, 36)	Inducing of apoptosis in activated CD4+ T cells (37)	Apoptosis/proliferation of inhibition of dendritic and B cells (26, 38)
Impact on cross-regulation model parameters	Decreased proliferation rate of Treg/Teff	Increased death rate of Treg/Teff	Decreased death rate of cAPC by T-cell killing

Different studies provided evidence for the presence of Tregs collected from healthy individuals with the capacity of recognizing and suppressing immune responses against these common viruses (Table 1) (27–29). To understand the putative role of Tregs on infection, a steady-state analysis of the extended cross-regulation model is performed under the assumption that these viruses are recognized by a single and viral-specific clone associated with a given cAPC. This assumption is in line with the notion that these viruses, although becoming chronic, do not usually lead to severe immunosuppression to the host, as predicted for viral infections with the capacity of eliminating all T-cell clones in the peripheral repertoire.

### Case I: HHV6

Infections by HHV6 are usually acquired early in life (24). The virus preferentially infects virus-specific CD4+ T cells inside which has the capacity of arresting cell cycle (35, 36). Infection latency is usually associated with macrophages only (24). To recreate this infection, the proliferation rates of Tregs and Teffs were reduced as function of the viral dynamics (Table 1). According to the respective steady-state analysis, four modes of infections emerge in this scenario.

**Figure 3:**
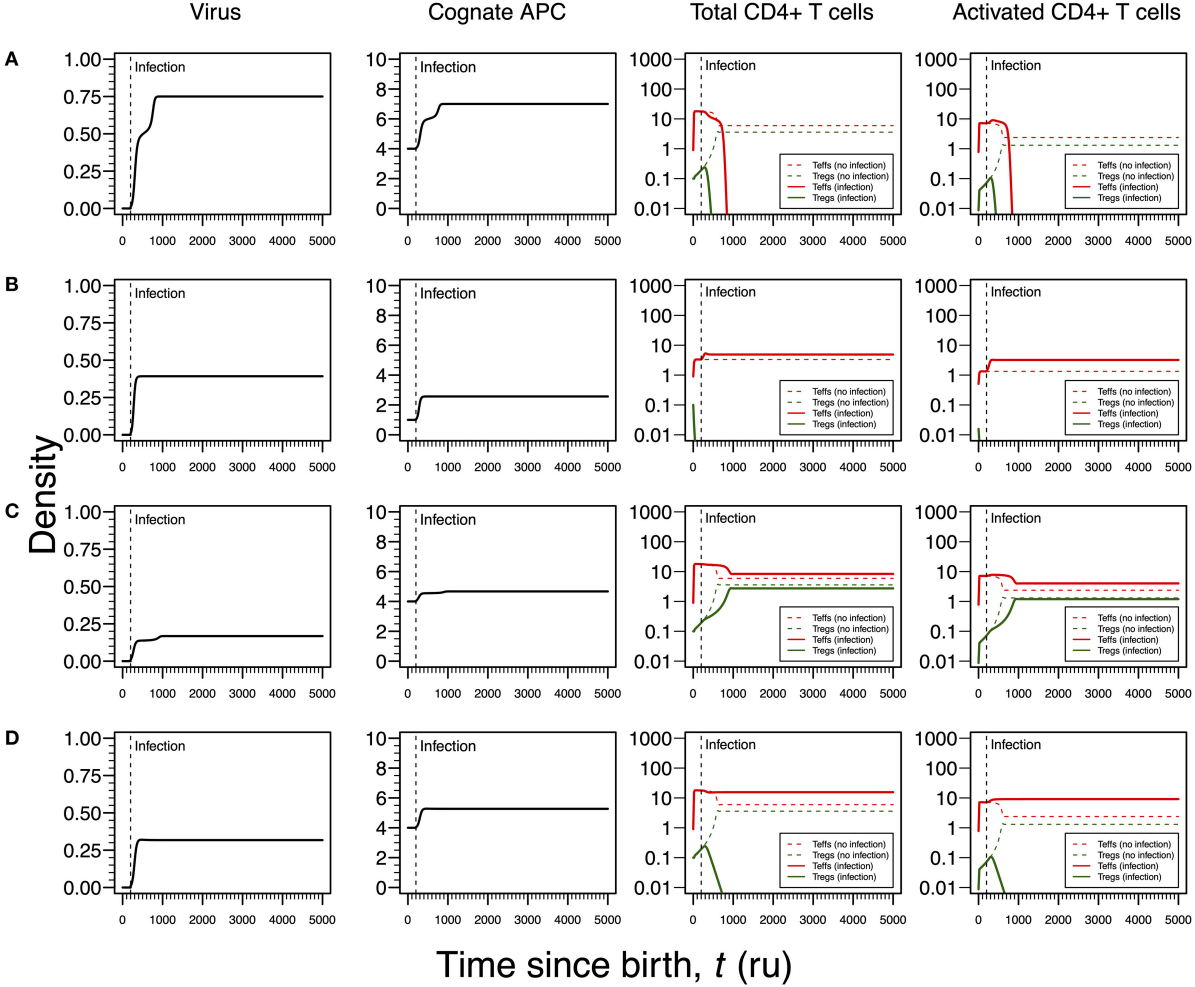
Four modes of HHV6 infection assuming an infection time at 200. HHV6 is assumed to decrease the T-cell proliferation rate as function of the viral density. T-cell dynamics in absence of infection were also included for comparison (dashed lines in T-cell plots). **(A)** Strong infection with a viral carrying capacity of 0.75 and recognized by a responding clone with a high autoimmune potential (A_0_ = 4). **(B)** Moderate infection with a viral carrying capacity of 0.45 and recognized by a responding clone with an intermediate autoimmune potential (A_0_ = 1). **(C)** Mild infection with a viral carrying capacity of 0.2 and recognized by a T-cell clone with a high autoimmune potential (A_0_ = 4). **(D)** Moderate infection with a viral carrying capacity of 0.5 and recognized by a T-cell clone with a high autoimmune potential (A_0_ = 4). The values for the remaining parameters can be found in Table S1.

The first infection mode is related to a high viral carrying capacity, which strongly reduces the proliferation rates of Tregs and Teffs of the virus-specific clone. This infection leads to a progressive reduction in the density of both Tregs and Teffs, which culminates with the extinction of the clone (Figure 3A). On the one hand, clonal extinction of the HHV6-specific clone, although allowing the virus to become chronic, might promote a deactivation of the infection due to the exhaustion of the virus-specific cells sustaining viral replication. If so, this type of infection might be interpreted as beneficial for the host and, thus, it could be associated with an individual who has a dormant or latent virus trapped inside macrophages. On the other hand, clonal extinction could lead to an uncontrolled HHV6 infection that, if occurring in the gut, might contribute to a systemic immune activation due to microbial translocation, as observed in HIV-infected individuals (46, 47). Such situation could ultimately promote immune deficiency, a condition not reported for ME/CFS. Therefore, this mode of infection can be interpreted as either a healthy or disease state but likely not related to ME/CFS *per se*.

The second infection mode is observed when the virus-specific clone has an intermediate autoimmune potential. In this case, Tregs of this clone become extinct before infection (owing to the fact that the respective abundance of cAPC was below the regulation threshold before infection). In theory, immune responses elicited by Teffs of this clone toward putative self-antigens are hypothesized to be under bystander suppression. The immune response is based on virus-specific Teffs alone (Figure 3B). On the one hand, these cells provide a cellular source for viral replication. On the other hand, an immune response driven by Teffs might generate helper signals that would recruit other immune cells like natural killer (NK) cells and CD8+ T cells to the infection site. If so, this type of infection can be interpreted as an immune response that has a lower chance of triggering either autoimmunity or ME/CFS.

The third infection mode occurs when the virus is recognized by a responding clone with a high autoimmune potential and the viral carrying capacity is low. In this case, the proliferation rates of the Tregs and Teffs from the responding T-cell clone are slightly affected by the virus. This leads to the maintenance of both cell types in the responding clone after infection (Figure 3C). In addition, the resulting steady states of Tregs and Teffs and their activated counterparts are increased when compared to a scenario of no infection. As above, the persistence of virus-specific T cells in an active immune response provides a continuous cellular source for viral replication. Since activated Tregs are known to have the capacity of suppressing the cytotoxic action of NK cells (48), then this mode of infection promotes a chronically active immune response under control of Tregs. This contributes to a continuous waste of energy and a persistent fatigue in the host. In this line of thought, this mode of infection might be then associated with ME/CFS, as hypothesized in this article.

The fourth mode of infection also involves the triggering of a responding clone with a high autoimmune potential, but alternatively associated with an intermediate viral carrying capacity (Figure 3D). In this case, the proliferation rates of Tregs and Teffs of the responding clone are moderately affected but such alteration is sufficient to induce the extinction of virus-specific Tregs. Since the responding clone has a high autoimmune potential, an immune response mounted solely by Teffs is not regulated and, therefore, it can be interpreted as the hallmark of an autoimmune disease resulting from an infection. If HHV6 is able to reach the brain and express a protein similar to the myelin basic protein (Table 1), then this mode of infection might initiate multiple sclerosis, an autoimmune disease whose clinical symptoms greatly overlap with the ones reported by many patients with ME/CFS (39). In this line of thought, the density of Tregs is expected to be affected by different viral capacities of the infecting HHV6, which may contribute to differences in pathogenesis and subsequent case definitions between multiple sclerosis and ME/CFS. It is important to note that persistent fatigue might be present in patients with different pathologies, including autoimmune diseases, cancer, or even eating disorders. However, chronic fatigue explained by any known disease, immune-mediated or not, is an exclusion criterion in current case definitions for ME/CFS diagnosis and research (1, 2). Therefore, the interpretations of the above infection modes might be biased according to these same case definitions.

### Case II: Herpes Simplex Virus Type I

In most cases, HSV1 is transmitted orally during childhood and/or adolescence (24). The main cellular targets of this virus are the skin epithelium cells and neurons during active and latent infections, respectively (24, 25). HSV1 is also known to induce apoptosis in infected monocytes and virus-specific CD4+ T cells (41). Given this line of evidence, the infection by this virus is then recreated in the cross-regulation model by an increase in the T-cell death rates as function of the viral dynamics (Table 1). Since increasing a death rate has a similar qualitative effect as decreasing a proliferation rate, the different modes of infections described for HHV6 can be again recreated for HSV1, but with some differences in the respective interpretations. The major difference between these two infections is the timing of the primary infection. In the case of HSV1, a later primary infection is more likely to occur when the responding clone might be already in equilibrium in absence of infection.

**Figure 4:**
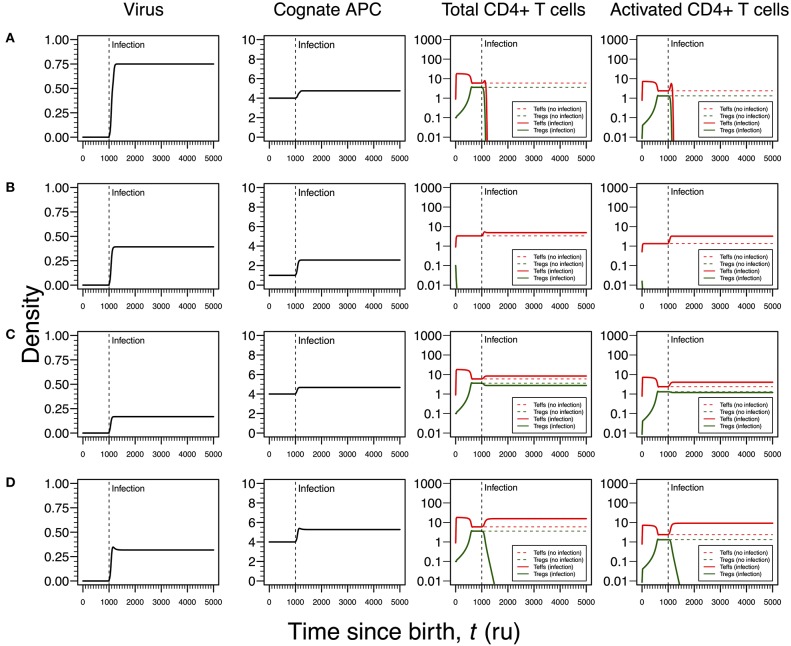
Four modes of HSV1 infection assuming an infection time at 1,000. HSV1 is assumed to increase the T-cell death rate as function of the viral density. T-cell dynamics in absence of infection were also included for comparison (dashed lines in T-cell plots). **(A)** Strong infection with a viral carrying capacity of 0.75 and recognized by a T-cell clone with a high autoimmune potential (A_0_ = 4). **(B)** Moderate infection with a viral carrying capacity of 0.45 and recognized by a T-cell clone with an intermediate autoimmune potential (A_0_ = 1). **(C)** Mild infection with viral carrying capacity of 0.2 and recognized by a T-cell clone with a high autoimmune potential (A_0_ = 4). **(D)** Moderate infection with a viral carrying capacity of 0.5 and recognized by a T-cell clone with a high autoimmune potential (A_0_ = 4). The values for the remaining parameters can be found in Table S1.

Similar to the infection mode shown in Figure 3A, an infection with a strong impact on the death rate of Tregs and Teffs implies a decrease in the density of Tregs and Teffs, which ultimately leads to the extinction of the responding clone (Figure 4A). However, since the main targets of HSV1 are the skin epithelium cells and neurons, this clonal extinction might lead to an uncontrolled infection only sustained by the cytotoxic action of other effector immune cells that do not require any helping signals from the CD4+ T cells. In a situation where these backup cells fail to sustain the infection, this immunological phenotype might be interpreted as a severe clinical condition, such as the herpetic stromal keratitis (49).

As illustrated in Figure 3B, the second mode of infection is associated with an intermediate viral carrying capacity and recognized by a responding clone with an intermediate autoimmune potential whose Tregs become extinct before infection (Figure 4B). Again, it is hypothesized that putative autoimmune responses induced by this clone before and during infection are under check by bystander suppression. This infection implies a moderate increase on death rate of Teffs of the responding clone and, therefore, these cells can persist during the immune response and be used as a cellular source for viral replication. However, activated Teffs are able to send signals and recruit other immune cells that could limit the pathology of this infection and promote viral latency. In this line of thought, this mode of infection has a low probability to degenerate into ME/CFS or an autoimmune disease.

Again, the remaining two modes of infection are associated with a responding clone with a high autoimmune potential. Acetylcholine receptor and α-synuclein are two possible human proteins with possible molecular mimicry with HSV1 proteins (31, 32). Similar to the situation recreated in Figure 3C, if the virus induces a mild increase in the death rates of Teffs and Tregs, then the respective immune response is characterized by a cross-regulation between these cell types (Figure 4C). As interpreted above for the HHV6 case, the persistence of CD4+ T cells in the immune response provides a cellular source for viral persistence, thus contributing to chronic active infection. More importantly, the densities of activated Teffs and Tregs are substantially increased in equilibrium (e.g., chronicity) when compared to absence of infection. This is consistent with a chronic inflammation and persistence fatigue. In contrast, if the infection is associated with an intermediate viral carrying (thus increasing the T-cell death rate moderately), then the respective immune response is mounted by Teffs only due to the extinction of Tregs in the same clone (Figure 4D). This mode of infection can be then linked to the onset of an autoimmune disease, such as myasthenia gravis (50).

### Case III: Epstein–Barr Virus

Infections by EBV are ubiquitous worldwide as evidenced by the high prevalence of antibodies against multiple EBV proteins in adults and the high frequency of healthy blood donors with detectable viral DNA (51). Primary infection is often asymptomatic in infants but frequently results in infectious mononucleosis in adolescents and adults (24). The preferred cellular targets are B cells in which the virus has the capacity of inhibiting proliferation or apoptosis (26). B cells are typically seen as antibody-producing cells but yet they have also an important role as antigen-presenting cells to CD4+ T cells due to the expression of major histocompatibility class II molecules (52). In this scenario, the effect of EBV infection is described by a T-cell killing of infected cAPC (Table 1). Again, four modes of infections emerge from the respective steady-state analysis.

**Figure 5:**
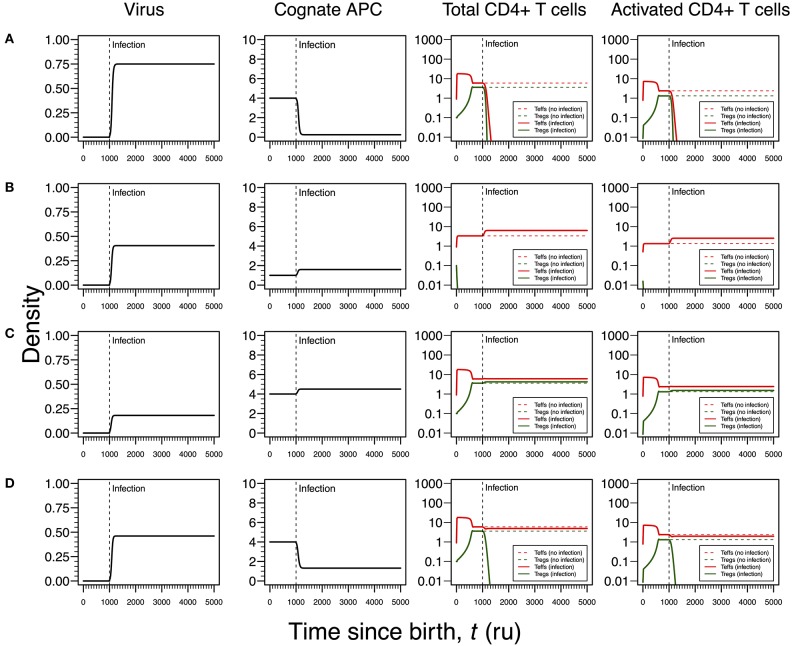
Four modes of EBV infection assuming an infection time at 1,000. EBV is assumed to have a direct impact on the dynamics of cAPC (in this case, B cells). T-cell dynamics in absence of infection were also included for comparison (dashed lines in T-cell plots). **(A)** Strong infection with a viral carrying capacity of 0.75, recognized by a T-cell clone with a high autoimmune potential (A_0_ = 4) and lack of T-cell capacity in killing infected cAPC. **(B)** Moderate infection with a viral carrying capacity of 0.45 and recognized by a T-cell clone with an intermediate autoimmune potential (A_0_ = 1) (T-cell killing rate of infected cAPC fixed at 0.1). **(C)** Mild infection with a viral carrying capacity of 0.2 and recognized by a T-cell clone with a high autoimmune potential (A_0_ = 4) (T-cell killing rate of infected cAPC fixed at 0.05). **(D)** Moderate infection with a viral carrying capacity of 0.5 and recognized by a T-cell clone with a high autoimmune potential (A_0_ = 4) (T-cell killing rate of infected cAPC fixed at 0.5). In plot **(A)**, the “growth” of cAPC as a function of the virus was set at −5. A negative growth can be interpreted as the direct killing of cAPC. For plots **(B–D)**, the growth of the cAPC as a function of the virus was fixed at 4. This means that the virus does not have the ability to kill the cAPC directly, allowing these cells to grow upon infection.

As shown in Figures 3A, 4A for HHV6 and HSV1, the first mode of infection is associated with a virus with the capacity of extinguishing the responding T-cell clone (Figure 5A). This only occurs when the virus has the capacity of killing cAPC directly in absence of any T-cell response against cAPC themselves. There is then a quick reduction of cAPCs, which are unable to maintain the responding clone. Again, clonal extinction might be interpreted as a situation of uncontrolled infection. However, the density of cAPCs (e.g., B cells) is very low in equilibrium, which in principle limits the quantity of viruses that can be reactivated during latency. In this line of thought, most of available viruses in this type of infection are likely to enter into a latency stage, thus not causing any persistent pathology.

Similar to Figures 3B, 4B, the second mode of infection induces an immune response of a clone with an intermediate autoimmune potential (Figure 5B). Tregs in this clone vanished before infection but autoimmune responses by the remaining Teffs might be avoided by bystander suppression. The immune response against EBV is then mounted by Teffs only. Again, an immune response based on Teffs only is likely to recruit other effector immune cells that ultimately would contribute to a reduction of the infection burden in the host. If so, this scenario can be then interpreted as an immune response that has a diminished chance to cause any pathology.

As illustrated in Figures 3C, 4C, the third mode of infection is associated with a responding clone with a high autoimmune potential and a low T-cell killing rate of cAPC (Figure 5C). This is a situation where the immune response against EBV is characterized by a cross-regulation between Teffs and Tregs. More importantly, the densities of Tregs and their activated counterparts at equilibrium (e.g., chronicity) are slightly increased when compared to absence of infection. Again, increased activation contributes to a chronic inflammation and persistent fatigue.

The fourth mode of infection is also associated with an immune response against EBV driven by a responding clone with a high autoimmune potential (Figure 5D). However, if Teffs are able to kill the infected APC at a moderate rate, this leads to a decrease in the density of cAPC. In turn, the decrease in the cAPC density promotes the extinction of Tregs in the responding clone. As for HHV6 and HSV1, this mode of infection might be associated with an autoimmune disorder.

### Common Infections Promote a Large Number of Combinations of Possible Regulated and Non-regulated Autoimmune Responses

**Figure 6:**
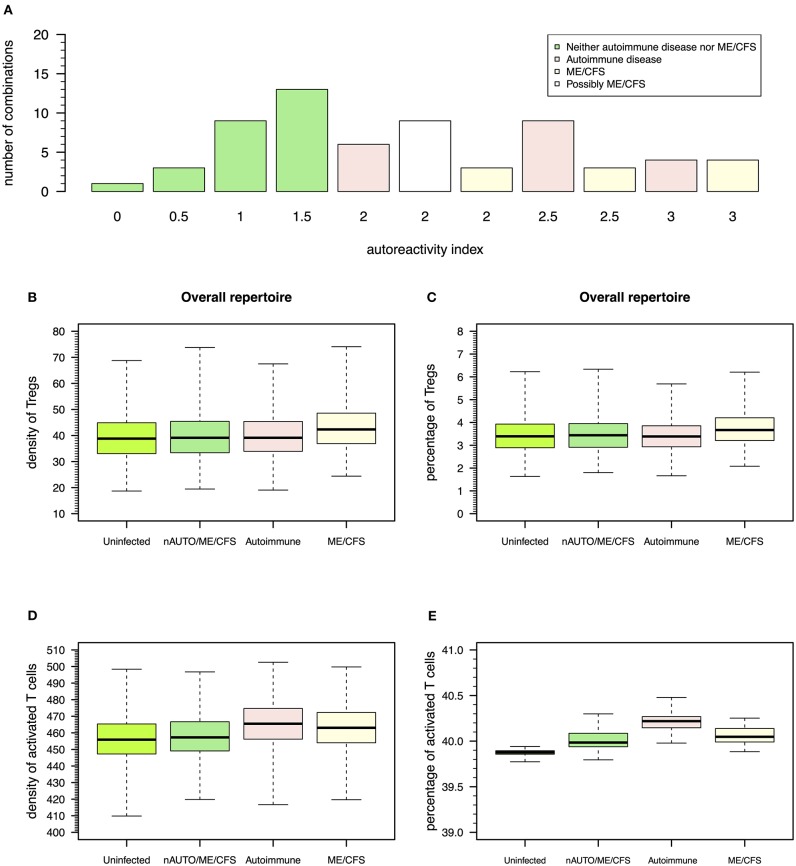
Possible definitions of patients with a putative autoimmune disease or with ME/CFS and the corresponding model simulations for densities of Tregs and activated T cells. **(A)** Frequency of possible combinations of qualitative immune responses against HHV6, HSV1, and EBV infections in the same individual that generate each possible value for autoreactivity index. Using this index, individuals who are neither an autoimmune nor a ME/CFS (nAUTO/ME/CFS) patient are defined by all combinations of infections that have an overall autoimmunity index ≤1.5. Individuals with either ME/CFS or a putative autoimmune disease are in turn associated with an autoreactivity index >1.5. Individuals with ME/CFS are defined by all combinations of infections where there is an equal or higher number of autoreactive responses under control of Tregs. Individuals with an autoimmune disease are defined otherwise. **(B,C)** Boxplots of the density and percentage of Tregs in simulated data from 500 uninfected healthy individuals, 500 infected nAUTO/ME/CFS individuals, 500 patients suffering from a putative autoimmune disease, and 500 patients suffering from ME/CFS. **(D,E)** Boxplots of the density and percentage of activated T cells in the same simulated data shown in **(B,C)**. The boxplots represent the minimum (lower whisker), the 25% quantile (lower hinge of the box), the median (central horizontal line within the box), the 75% quantile (upper hinge of the box), and the maximum (upper whisker) of the simulated data.

The above steady-state analyses showed four qualitative modes of infection for each virus. Since most individuals are known to be exposed to all these viruses during their lifetime, it is important to consider all possible combinations of qualitative immune responses against HHV6, HSV1, and EBV infections that can occur in the same individual. There is then a total of 64 (= 4^3^) possible combinations of modes of infection for these three viral infections in the same individual. To help interpret these combinations in terms of health, autoimmune disease, and ME/CFS, the qualitative interpretation of the immune response against each viral infection is scored as follows: 0 for a mode of infection where the responding clone go extinct, 0.5 for a mode of infection triggering the response of a clone with a moderate autoimmune potential, and 1 for a mode of infection triggering the response of a clone with a high autoimmune potential, as illustrated in Figures 3–5. These scores for individual infections in the same individual allow the generation of an overall autoreactivity index for each one of the 64 combinations by summing the autoreactivity potential of the modes of infection associated with each virus (Table S2). The possible values for this autoreactivity index are 0, 0.5, 1, 1.5,…, up to 3, where 0 indicates a combination of the three infections in the same individual where all responding clones would go extinct, and 3 indicates combinations of the three infections in the same individual, where each virus induced the response of a clone with a high autoimmune potential (under or not Treg regulation). Assuming a cumulative effect of these infections on a fatigue scale or fatigue persistence in a particular individual, an overall autoreactive index higher than 1.5 indicates a predominance of infections triggering autoimmunity in the same individual, therefore being more than associated with ME/CFS and a putative autoimmune disease. In this line of thought, there are 26 combinations of immune responses in the same individual without an autoimmune pathology or ME/CFS (Figure 6A). The remaining 38 combinations can then be associated with either an autoimmune disease or ME/CFS. For a matter of simplicity, an autoimmune disease is here defined by all combinations of infection modes in the same individual where there is a higher number of immune responses against these viruses, mounted by autoreactive Teffs under no regulation of Tregs (*n* = 19; Figure 6A). In contrast, ME/CFS is defined as a predominance of immune responses to these viruses exerted by clones with autoimmune potential but under regulation of Tregs. There are 10 combinations of immune responses against the three viruses under this condition (Figure 6A). Finally, there are nine combinations of immune responses in the same individual, where there is an even number of individual autoreactive immune responses regulated and non-regulated. These combinations, although undefined in terms of a predominance of cross-regulation or autoimmunity, are likely associated with chronically active infections. On the one hand, chronic activation of the immune system is likely to cause fatigue. On the other hand, persistent active infections are likely to manifest themselves by a higher frequency of symptoms related to viral infections often reported by patients with ME/CFS. In this line of thought, these combinations of immune responses can be seen as part of the large spectrum of symptoms presented by patients with ME/CFS.

### Quantitative Predictions for the Number of Tregs in Patients Suffering From ME/CFS

After defining which combinations of infection modes are more consistent with each clinical condition, the cross-regulation model can be used to make predictions for the density of Tregs and Teffs in healthy controls, patients suffering from a putative autoimmune disease, or patients suffering from ME/CFS. These predictions are useful for data interpretation given the lack of a reliable biomarker for ME/CFS.

For illustrative purposes, a simulation of the CD4+ T-cell repertoire was performed for 500 uninfected healthy controls, 500 infected individuals who do not suffer from an autoimmune disease or ME/CFS, 500 infected patients suffering from an autoimmune disease, and 500 infected patients suffering from ME/CFS. A detailed description of the simulation procedure can be found in Supplementary File.

According to this simulation, patients suffering from ME/CFS had, on average, a slightly higher density and percentage of Tregs than individuals from the remaining groups (Figures 6B,C and Table S3). Therefore, the definition used for ME/CFS is consistent with available data from the clinic. On average, there was a higher density of activated T cells in patients with either ME/CFS or a putative autoimmune disease when compared to healthy controls (Figure 6D). However, when the respective cell densities were converted into percentages, all groups had a similar average percentage of activated T cells (Figure 6E). This result suggested that the crude immunological analysis based on percentage data alone might mask potentially relevant differences between healthy controls and patients suffering from ME/CFS.

## Concluding Remarks

This article discussed the overlooked role of Tregs on the pathology of ME/CFS using the cross-regulation model and its variant for viral infections. In particular, the discussion focused on the impact of HHV6, HSV1, and EBV infections, as these viruses are highly prevalent in human populations, thus being major candidates for triggering ME/CFS. Other viruses such as herpes simplex virus 2 and cytomegalovirus have also been reported as alternative disease triggers (4). However, since the prevalence of these lesser common viruses vary considerably across countries (53–55), they might represent triggers of ME/CFS in specific populations. This highlights the difficulty of identifying a master infection other than HHV6, HSV1, and EBV that could trigger ME/CFS. In this line of thought, the onset of both ME/CFS and a given autoimmune disease was hypothesized as the result of a cumulative effect of autoreactive immune responses to these common viral infections. In particular, the pathogenesis of ME/CFS was hypothesized as a predominance of antiviral responses by autoreactive clones under control of Tregs. This hypothesis is quantitatively translated into a high density and percentage of Tregs in patients with ME/CFS when compared to healthy controls and patients with a putative autoimmune disease, as illustrated with model simulations and real data from the clinic (15–17). In addition, an increased regulation by Tregs might also lead to reduced microbial immunity in patients with ME/CFS. This expectation is also in agreement with the frequent viral infections reported by patients suffering from this disease (3). In the same line of evidence, a recent study found defective T-cell responses to EBV in patients of this disease (56).

For discussing the role of Tregs on ME/CFS, simplifying assumptions were made for the construction of the cross-regulation model. The most important one was to not distinguish the dynamics of natural and inducible Tregs. Therefore, the discussion could only be done for Tregs as a whole. In theory, modeling the dynamics of natural and induced Tregs is expected to improve the insights into ME/CFS. It is known that these two cell types have distinct biology and molecular requirements for their function and cell survival (57). Natural Tregs require co-stimulation with CTLA-4 and are typically associated with IL-2 expression, whereas inducible Tregs typically exert their regulatory function in immunological niches enriched with TGFβ. Given these observations, a more detailed extension of the cross-regulation model could be used not only to investigate the dynamics of natural and induced Tregs during a viral infection but also to determine the expected relationship with the resulting cytokine expression profiles. This could help to identify specific cytokine signatures associated with ME/CFS. Until now, the most reported cytokine in ME/CFS literature is TGFβ, whose expression tends to be increased in patients suffering from this disease (18, 19). A systematic review also provided some evidence for an increased IL-2 expression in patients with ME/CFS, but most of these studies were statistically inconclusive (18). In face of these different studies, ME/CFS could result from an increased regulation of induced Tregs supported by active HHV6, HSV1, and EBV infections. A way to test this hypothesis is to investigate a possible molecular mimicry between viral and human proteins given that Tregs are thought to target tissue antigens. Then, the existence of specific Tregs recognizing such target proteins should be assessed in patients suffering from ME/CFS and compared with healthy controls. If so, healthy controls are expected to have a lower fraction of these specific cells within the Treg pool than patients suffering from ME/CFS. This type of investigation has already been attempted for type I narcolepsy, an autoimmune disease that can be triggered by a molecular mimicry between the human hypocretin protein and proteins from H1N1 influenza A virus (58). In agreement with the proposed hypothesis, patients suffering from this autoimmune disease tend to have, on average, an increased number of Tregs than healthy controls (59). With respect to ME/CFS, a putative molecular mimicry was recently suggested between an EBV-derived peptide (EBNA6), human thyroid peroxidase, and other human proteins (60). However, it is still unclear whether other viruses associated with ME/CFS (4) would induce similar molecular mimicry with tissue antigens.

In this discussion, the CD4+ T-cell repertoire was assumed to be composed of independent clones, each one of them with a specific dynamic determined by a specific cAPC available at a given abundance in the periphery (20). In theory, these independent clones are required to recognize non-overlapping sets of antigens through their T-cell receptors or to interact with mutually exclusive sets of cAPCs. This assumption is unrealistic for non-specific antigen presentation provided by macrophages and dendritic cells. However, specific antigen presentation is less likely to occur in viral infections where a more specialized immune response might be required. In the context of infections, B cells are known not only to produce antibodies involved in pathogen clearance, but also to enhance ongoing immune response by performing highly specialized antigen presentation to CD4+ T cells (52). In this scenario, the assumption of independent T-cell clones with distinct sets of cAPCs can be re-interpreted as a possible interaction between two highly specific cellular repertoires, one related to CD4+ T-cells and another related to B cells serving as their cAPCs. If so, the theory based on cross-regulation model can help interpret the potential effects of current therapies based on B-cell depletion on patients with ME/CFS or with multiple sclerosis (60–63). The basic idea of these therapies is to eliminate B cells in order to have a re-population of the periphery with a new B-cell repertoire generated from the bone marrow. These therapies then induce a reduction of specific antigen presentation to CD4+ T cells carried out by B cells. If this reduction affects all B-cell clones irrespective of their antigen specificity, then the distribution of cAPC density associated with a given individual (Figure 2B) is expected to shrink upon treatment. According to the cross-regulation model, such therapeutic reduction in cAPC density would promote a slow depletion of Tregs together with a reduction in the number of Teffs belonging to the same highly autoreactive clones. If ME/CFS is indeed related to a hyperregulation of CD4+ T-cell responses toward common viruses, then this depletion is expected to bring some benefits to the respective patients. On the other hand, a reduction in antigen presentation by B cells might also lead to a depletion of many T-cell clones with an intermediate autoimmune potential in which the resulting density of their cAPC would go below the survival threshold during treatment. If these clones are indeed the main source of most immune responses, as hypothesized elsewhere (20), their depletion might pose a threat to patients if they experience any infection during treatment. In addition to these effects, the success rate of treatments based on B-cell depletion might require understanding the interplay between the rate by which new B cells can repopulate the periphery, the frequency by which a drug is administrated and the rate by which individuals are normally infected. This interplay should be investigated in future clinical trials.

Another simplifying assumption in this discussion was to neglect the impact of a continuous thymic export to the periphery. In theory, thymic export influences the T-cell repertoire by introducing new clones into periphery. Given the high combinatorial nature of the genetic rearrangement mechanism encoding the T-cell receptor, these new clones should exhibit different antigen specificities from the ones already existing in the periphery. Interestingly, early work using a more complex cross-regulation model demonstrated that most of the clonal selection shaping the T-cell repertoire occurs early in life (21). After this time period, the resulting T-cell repertoire is enriched with resident clones at sufficiently high density. These resident clones tend to be highly autoreactive and are able to outcompete newly generated clones typically at lower densities. In this scenario, the role of thymic export is only relevant to understand the impact of infections targeting clones with an intermediate autoimmune potential. Since an autoimmune disease and ME/CFS were here associated with clones with a high autoimmune potential, the above results should not be affected by the inclusion of a thymic output in the cross-regulation model. A possible limitation of excluding a thymic export from the model is related to a putative re-activation of dormant HHV6, HSV1, and EBV infections that could trigger ME/CFS. Viral dormancy was here interpreted as resulting from infections with the potential of extinguishing the responding clone. This interpretation seems particularly reasonable for HHV6 and HSV1, two viruses associated with CD4+ T-cell tropism. In these cases, if the thymus generates a new clone with similar HHV6 and HSV1 antigen specificity, then this clone might serve as a renewed cellular source for viral replication. This event might then promote the re-activation of the existing dormant infections. Again, the high combinatorial nature of the T-cell receptor rearrangement mechanism suggests that the re-activation of dormant infection via the generation of new clone with similar antigen specificity should be a very unlikely event in individuals without any genetic abnormality underlying the generation of the T-cell repertoire. Interestingly, genetic polymorphisms on the locus encoding the alpha chain of the T-cell receptor were found to be associated with ME/CFS (64). However, the study of the T-cell receptor repertoires in patients with ME/CFS remains to be performed and, therefore, it is still unclear whether these genetic polymorphisms are indeed in the true causal pathway of the disease.

A final simplification of this discussion was to assume a single responding clone against each virus. This simplification is in line with the concept of immunodominance where only a fraction of possible epitopes is able to stimulate a T-cell response (65). Immunodominance can also be defined as the frequency of T cells targeting individual antigens (66). This is a very useful concept, because it restricts the analysis to a small number of viral antigens and clones. For example, lytic cycle antigens are possible immunodominant targets of CD4+ T cells against EBV infections (67). In reality, a given infection is likely to trigger a polyclonal immune response targeting different viral proteins. This was suggested by a recent study in which the antibody levels against different EBV-derived peptides were quantified in patients with ME/CFS (68). In theory, these polyclonal immune responses can be simulated using the cross-regulation model under the assumption of independent clones, where each clone recognizes a given viral protein. However, the simulation of such a polyclonal immune response, although interestingly, seemed more appropriate for a study with the specific aim of generating predictions for the shape of the CD4+ T-cell repertoire in ME/CFS patients, which was out of the scope of this work.

It is worth noting that the cross-regulation model was the first one to be developed to understand the immune-physiology of Tregs specifically (23). Since its first formulation, this model has been used to explain the hygiene hypothesis for autoimmune diseases and to tackle tumor immunobiology (69, 70). Other mathematical models for the dynamics of Tregs also emerged in the literature, as reviewed in detail elsewhere (71). They were formulated under different mechanisms, such as APC maturation and direct suppression of Teffs by Tregs. They offer then alternative avenues of research to unravel the impact of Tregs on the pathology of ME/CFS. In the context of a specific disease, a mathematical model of Tregs dynamics was proposed for studying multiple sclerosis in humans and experimental autoimmune encephalomyelitis in lab mice (72, 73). In contrast with the cross-regulation model, this model described tissue damage explicitly as well as its relationship with the quantity of antigen presentation to CD4+ T cells. This feature of the model led to a stable oscillatory behavior for the respective dynamics, thus providing a mechanistic explanation for symptoms relapses typically observed in patients with multiple sclerosis. Similar relapsing–remitting symptoms are typically observed in patients with ME/CFS (74) and, therefore, this alternative model might be redeployed to investigate this disease and its putative differences with multiple sclerosis.

In conclusion, this discussion is a first attempt to tackle the pathology of ME/CFS from a mathematical modeling point of view. It hopes to foster research of this disease beyond a collection of disparate evidence where disease causality is difficulty to be ascertained due to possible selection bias and disease misclassification (75). In this regard, mathematical modeling under realistic assumptions provides invaluable theory upon which different hypotheses can be tested. In this article, the focus of discussion was the specific impact of Tregs in ME/CFS using a well-established mathematical model. Alternative hypotheses concerning the role of other immune-cell populations in ME/CFS are welcome but, bearing in mind, they can be rigorously assessed by developing an appropriate mathematical model. In this regard, mathematical modeling of the immune system has a sufficiently long history to provide ME/CFS researchers an already-built mathematical model tailored to their needs (76). As such, mathematical modeling shows promise to accelerate the research and treatment of this complex disease.

## Data Availability Statement

The datasets generated for this study are available on request to the corresponding author.

## Author Contributions

NS conceptualized this research. NS and JC developed the cross-regulation model and its extension to chronic viral infections, implemented the mathematical models, and performed all computer simulations. EL and LN helped interpret and discuss all results. All authors have read, revised, and approved the final draft of the manuscript.

### Conflict of Interest

The authors declare that the research was conducted in the absence of any commercial or financial relationships that could be construed as a potential conflict of interest.

## Abbreviations

cAPC: cognate antigen-presenting cell
EBV: Epstein–Barr virus
HHV6: Human Herpes virus 6
HSV1: Human simplex virus 1
ME/CFS: myalgic encephalomyelitis/chronic fatigue syndrome
TGFβ: transforming growth factor beta
Treg: regulatory T cell
Teff: effector T cell.
