# Supplementary material for: Myalgic Encephalomyelitis/Chronic Fatigue Syndrome as a Hyper-Regulated Immune System Driven by an Interplay Between Regulatory T Cells and Chronic Human Herpesvirus Infections
Source: Front Immunol. 2019 Nov 21;10:2684. doi: 10.3389/fimmu.2019.02684 (PMC6883905; doi:10.3389/fimmu.2019.02684)
Supplement: Supplementary file 1 [file Data_Sheet_1.pdf]

Supplementary Material

1 THE BASIC CROSS-REGULATION MODEL IN ABSENCE OF INFECTION

The model describes the dynamics of the density of Teffs E and the density of Tregs R in a clone of T cells by the following set of differential equations:

$$\frac{dE}{dt} = \pi_e E_A - \delta_e E \; and \; \frac{dR}{dt} = \pi_r R_A - \delta_r R, \qquad [1]$$

where E_A and R_A are the densities of activated Teffs and Tregs, respectively, π_e and δ_e are the proliferation and death rates of Teffs, respectively, and π_r and δ_r are the proliferation and death rates of Tregs, respectively. The dynamics of T-cell activation is modelled as the result of productive conjugations between these cells and their cognate APC, whose density is denoted by A. As an approximation, the processes of conjugation and deconjugation is assumed to happen at a faster time scale than the one governing changes in the T-cell density. Under this assumption, the density of conjugates is assumed to be in quasi-steady and one can calculate the total density of conjugated T cells as:

$$C = \frac{1+\kappa(sA+T)-\sqrt{(1+\kappa(sA+T))^2-4sAT\kappa^2}}{2\kappa}, \qquad [2]$$

where $T = E + R$ and κ is the conjugation constant (i.e., the ratio between the conjugation and deconjugation rates), and s is the number of conjugation sites per APC (assumed to be 2 here). A detailed derivation of this formula can be found elsewhere (1).

The density of conjugated Teffs (E_c) and Tregs (R_c) is assumed to be proportional to the relative density of each cell type at a given time, i.e.,

$$E_c = \frac{E}{E+R} C \text{ and } R_c = \frac{R}{E+R} C. \qquad [3]$$

In turn, these cellular densities are used to calculate fraction of APC conjugation sites that are occupied by Teffs and Tregs as follows

$$\theta_e = \frac{E_c}{A} \text{ and } \theta_r = \frac{R_c}{A}. \qquad [4]$$

The densities of activated Teffs (E_A) and Tregs (R_A) are finally obtained from the respective conjugated density based on the following functions:

$$E_A = E_c\left(1 - \frac{2\theta_r}{2-\theta_e}\right) \text{ and } R_A = R_c\left(\frac{2\theta_e}{2-\theta_r}\right). \qquad [5]$$

These are *ad hoc* functions which have been chosen to capture the complex non-linear interactions between Teffs and Tregs in multicellular conjugates, originally described in Leon et al. (1). These functions allow the model with two conjugation sites to display bi-stability as well as bifurcations and modulation of the basins of attraction of the steady states comparable to those obtained with higher numbers of conjugation sites.

2 EXTENDED CROSS-REGULATION MODEL TO DESCRIBE CHRONIC VIRAL INFECTIONS

The above cross-regulation model is here extended to the scenario where a viral infection occurs at a given instant τ. The main objective for constructing this extended model is to provide a quantitative framework where the role of Tregs on ME/CFS can be discussed. With this objective, the dynamics of the responding T-cell clone is firstly described by the basic cross-regulation model from birth (t=0) to

the instant τ. Then, the dynamics of the responding T-cell clone and its cAPC are changed by the infection. Since infections by HHV6, HSV1 and EBV are chronic in human populations, the viral density I is described by a logistic growth curve with carrying capacity γ and growth rate ω, which is affected by an inhibitory interaction term with activated Teffs associated with viral "death" rate δ_v:

$$\frac{dI}{dt^*} = \omega I\left(1 - \frac{I}{\gamma}\right) - \delta_v I E_A \quad [5]$$

where $t^* = t - \tau$. Note that $I(t)$=0 for t<τ. For simplicity, the parameter γ is normalized between 0 and 1, where 1 is the maximum viral carrying capacity of a given individual. In turn, the dynamics of cAPC A is assumed to follow the differential equation

$$\frac{dA}{dt} = s - \delta_A A + wI - \rho I E_A, \quad [6]$$

where s represents a constant influx of cAPC, δ_A is the death rate of cAPC in absence of infection, w is the differentiation rate from monocytes to cAPC upon infection, and ρ is the removal rate of infected APC by the action of activated Teffs. For a matter of simplicity, the dynamics of cAPC was assumed to be quasi-steady as function of I, which leads to the following simplification

$$A(t) = A_0 + \theta I(1 - k_A E_A), \quad [7]$$

where $A_0 = s/\delta_A$, $\theta = w/\delta_A$ and $k_A = \rho/w$.Note that θ reflects the ratio between differentiation rate promoted by the virus and "natural" death rate of APC. In practice, this ratio can be set as a negative value, which can be interpreted as the capacity of the virus in directly killing cAPC (i.e., a negative value for w).

Since CD4+ T cells are main targets of HHV6 and HSV1, it is assumed to these cells would not remove their cAPC upon conjugation. As a consequence, the parameter k_A is set to zero for these infections. The resulting dynamics is given by the following equation

$$A(t) = A_0 + \theta I. \quad [8]$$

The dynamics of the T-cell clone responding to the infections follows equation [1] but now the proliferation and death rates of Teffs and Tregs are described as functions of the viral density (i.e., $\pi_e^*(I)$, $\pi_r^*(I)$, $\delta_e^*(I)$ and $\delta_r^*(I)$). Briefly, these functions were chosen either to be constant (EBV), to reduce T-cell proliferation rate (HHV6) or to increase T-cell apoptosis in the case (HSV1), as explained below.

The functions $f(I), \pi_e^*(I)$, $\pi_r^*(I)$, $\delta_e^*(I)$ and $\delta_r^*(I)$ are here chosen to describe biologically plausible impacts of HHV6, HSV1, and EBV on the dynamics of the responding T-cell clone and its cAPC. With respect to HHV6, this virus preferentially infects CD4+ T cells reducing their proliferation rate (24,25). Then, $\pi_e^*(I) = \pi_e(1 - I)$, $\pi_r^*(I) = \pi_r(1 - I)$, $\delta_e^*(I) = \delta_e$ and$\delta_r^*(I) = \delta_r$, where π_e, π_r, δ_e, and δ_r are the parameters associated with the basic formulation of the cross-regulation model. HSV1 also infects CD4+ T cells but instead increasing their death rate (26). As a consequence, $\delta_e^*(I) = \delta_e/(1 - I)$, $\delta_r^*(I) = \delta_r/(1 - I)$, $\pi_e^*(I) = \pi_e$ and $\pi_r^*(I) = \pi_r$. Finally, EBV is assumed to only the proliferation of B cells, which can also be considered as highly specific cAPC (29). In this case, $\pi_e^*(I) = \pi_e$, $\delta_e^*(I) = \delta_e, \pi_r^*(I) = \pi_r$ and $\delta_r^*(I) = \delta_r$.

3 MODEL PARAMETRIZATION

To discuss the putative role of Tregs on ME/CFS, the density of cAPC associated with a given T-cell clone is considered as a fixed parameter in the basic Cross-regulation model, which can be varied to perform a steady-state analysis. Under the assumption of a typical infection, the growth rate of the virus was assigned a reference value of 1, as done elsewhere (2,3).

With respect to the parameters governing CD4+ T-cell dynamics, the proliferation rate of Teffs was considered half of the one of the respective cAPC. Since it has been observed that about 10% of Teffs

can be converted into Tregs upon antigen stimulation (4), the proliferation rate of Tregs is assumed to be increased in the same amount in relation to the one of Teffs. The corresponding death rates were set at 0.2, as done elsewhere (3). This value accounts for 40% and 36% of the proliferation rate of Tregs, respectively. These percentages are in line with the relative death rates of CD4+T cells estimated from *in vitro* stimulation experiments (5,6).
As mentioned above, the possible values for the viral capacity at chronicity is assumed to be normalized from 0 to 1. In this scenario, this limit can be interpreted as the proportion of a hypothetical maximum viral density that any given host could sustain. In the case of the remaining parameters related to APC dynamics, they were varied accordingly to obtain different steady states.
A summary of the model parameters and their values are presented in Table S1. Since model parameterization is here specified as function of a reference value for the proliferation rate of cAPC, the time scale of the model simulations should be seen as proportional to this value.

TABLE S1. Summary of model parameters and their reference values.

Cell type	Parameter	Interpretation	Value
cAPC	A_0	Density prior to infection	$(0,\infty)$
	κ	Conjugation/deconjugation rate with T cells	1
	s	Number of conjugation sites per APC	2
	θ	Proportionality constant related to viral dynamics	$(-\infty,+\infty)$
	k_A	Ratio between removal "rate" of infected cAPC by Teffs and differentiation rate from monocytes to cAPC	0 (HHV6/HSV1) or $(0,+\infty)$ (EBV)
Teffs	π_e	Proliferation rate	0.5
	δ_e	Death rate	0.20
	E_0	Initial density	0.90
Tregs	π_r	Proliferation rate	0.55
	δ_r	Death rate	0.20
	R_0	Initial density	0.10
Virus	γ	Viral carrying capacity in the host	[0,1]
	ω	Time by which half of γ is achieved	0.05
	δ_v	Viral death rate by the action of activated Teffs	0.002

4 SIMULATION OF CD4+ T-CELL REPERTOIRE FOR DIFFERENT GROUPS OF INDIVIDUALS

A simulation of CD4+ T-cell repertoires was performed for 500 uninfected healthy controls, 500 chronically infected individuals who are neither autoimmune nor ME/CFS patients (nAUTO/ME/CFS), 500 chronically infected patients suffering from a putative autoimmune disease, 500 chronically infected patients suffering from ME/CFS. The repertoire of each individual was assumed to be composed of 100 independent CD4+ T-cell clones whose 97 clones do not recognize none of HHV6, HSV1 and EBV infections. The dynamics of these clones was solved for the reference values Table S1 using the basic cross-regulation model in absence of infection. The associated APC density was randomly taken from the Gamma distribution shown in Figure 2B with the restriction that 94 and 3 clones would have an associated cAPC density below and above the regulation threshold, respectively. The remaining 3 clones were assumed to recognize each virus specifically upon infection. For uninfected healthy individuals, the dynamics of these clones were randomly selected from one of the four ones shown in Figures 3, 4 and 5 in absence of infection. For the remaining individuals who are all assumed to be chronically infected by HHV6, HSV1 and EBV, it was simulated a random combination of infection modes shown in Figure 3, 4 and 5 in presence of different viral infections. A list of these combinations and the respective immunological phenotype can be found in Table S2. A statistical summary of the simulated repertoires in each group is provided in Table S3.

Table S2. Autoreactivity index and the respective clinical phenotype for each of 64 possible combinations of immune responses to HHV6, HSV1 and EBV infections in the same individual where nAuto/MECFS represents a phenotype that is neither the one of a patient with an autoimmune disease nor the one of a patient with ME/CFS.

HHV6	HSV1	EBV	Autoreactivity index	Phenotype
Clonal extinction	Clonal extinction	Clonal extinction	0	nAuto/ME/CFS
		Teff response	0.5	nAuto/nMECFS
		Autoimmunity	1	nAuto/nMECFS
		Regulation	1	nAuto/nMECFS
	Teff response	Clonal extinction	0.5	nAuto/nMECFS
		Teff response	1	nAuto/nMECFS
		Autoimmunity	1.5	nAuto/nMECFS
		Regulation	1.5	nAuto/nMECFS
	Autoimmunity	Clonal extinction	1	nAuto/nMECFS
		Teff response	1.5	nAuto/nMECFS
		Autoimmunity	2	Autoimmune disease
		Regulation	2	MECFS
	Regulation	Clonal extinction	1	nAuto/nMECFS
		Teff response	1.5	nAuto/nMECFS
		Autoimmunity	2	ME/CFS
		Regulation	2	ME/CFS
Teff response	Clonal extinction	Clonal extinction	0.5	nAuto/nMECFS
		Teff response	1	nAuto/nMECFS
		Autoimmunity	1.5	nAuto/nMECFS
		Regulation	1.5	nAuto/nMECFS
	Teff response	Clonal extinction	1	nAuto/nMECFS
		Teff response	1.5	nAuto/nMECFS
		Autoimmunity	2	Autoimmune disease
		Regulation	2	ME/CFS
	Autoimmunity	Clonal extinction	1.5	nAuto/nMECFS
		Teff response	2	Autoimmune disease
		Autoimmunity	2.5	Autoimmune disease
		Regulation	2.5	Autoimmune disease
	Regulation	Clonal extinction	1.5	nAuto/nMECFS
		Teff response	2	ME/CFS
		Autoimmunity	2.5	Autoimmune disease
		Regulation	2.5	ME/CFS
Autoimmunity	Clonal extinction	Clonal extinction	1	nAuto/nMECFS
		Teff response	1.5	nAuto/nMECFS
		Autoimmunity	2	Autoimmune disease
		Regulation	2	ME/CFS
	Teff response	Clonal extinction	1.5	nAuto/nMECFS
		Teff response	2	Autoimmune disease
		Autoimmunity	2.5	Autoimmune disease

		Regulation	2.5	Autoimmune disease
	Autoimmunity	Clonal extinction	2	Autoimmune disease
		Teff response	2.5	Autoimmune disease
		Autoimmunity	3	Autoimmune disease
		Regulation	3	Autoimmune disease
	Regulation	Clonal extinction	2	ME/CFS
		Teff response	2.5	Autoimmune disease
		Autoimmunity	3	Autoimmune disease
		Regulation	3	ME/CFS
Regulation	Clonal extinction	Clonal extinction	1	nAuto/nMECFS
		Teff response	1.5	nAuto/nMECFS
		Autoimmunity	2	ME/CFS
		Regulation	2	ME/CFS
	Teff response	Clonal extinction	1.5	nAuto/nMECFS
		Teff response	2	ME/CFS
		Autoimmunity	2.5	Autoimmune disease
		Regulation	2.5	ME/CFS
	Autoimmunity	Clonal extinction	2	ME/CFS
		Teff response	2.5	Autoimmune disease
		Autoimmunity	3	Autoimmune disease
		Regulation	3	ME/CFS
	Regulation	Clonal extinction	2	ME/CFS
		Teff response	2.5	ME/CFS
		Autoimmunity	3	ME/CFS
		Regulation	3	ME/CFS

TABLE S3. Summary statistics of the density and percentage of Tregs and activated CD4+ T cells from simulated repertoire of 100 independent clones from 500 uninfected individuals (Uninfected), 500 infected individuals who are neither autoimmune or ME/CFS patients (nAuto/ME/CFS), 500 infected patients with a putative autoimmune disease (Autoimmune) and 500 infected patients with ME/CFS (ME/CFS).

Analysis	Group	Mean (range)
Density of Tregs	Uninfected	39.6 (18.7-68.8)
	nAuto/ME/CFS	39.8 (19.5-73.8)
	Autoimmune	39.8 (19.1-67.5)
	ME/CFS	43.2 (24.4-74.1)
Percentage of Tregs	Uninfected	3.5 (1.6-6.2)
	nAuto/ME/CFS	3.5 (1.8-6.3)
	Autoimmune	3.4 (1.6-5.7)
	ME/CFS	3.7 (2.1-6.2)
Density of activated cells	Uninfected	456.0 (409.8-498.4)
	nAuto/ME/CFS	457.5 (419.8-496.8)
	Autoimmune	465.3 (416.7-502.6)
	ME/CFS	462.7 (419.7-499.7)
Percentage of activated cells	Uninfected	39.9 (39.8-40.0)
	nAuto/ME/CFS	40.0 (39.8-40.3)
	Autoimmune	40.2 (40.0-40.5)
	ME/CFS	40.1 (39.9-40.3)

5 COMPUTATIONAL IMPLEMENTATION

The numeric solutions of the basic cross-regulation model and its infection dynamics extension was performed using the package deSolve for the R software. The model was solved using the “vode” method, which uses a variable step. A sufficiently long simulation (e.g., t=25,000) was used to determine the steady states of a given clone in absence of infection. The respective scripts are freely available from the corresponding author upon request.

REFERENCES

1. León K, Peréz R, Lage A, Carneiro J. Modelling T-cell-Mediated Suppression Dependent on Interactions in Multicellular Conjugates. *J Theor Biol* (2000) **207**:231–254. doi:10.1006/jtbi.2000.2169

2. Fouchet D, Regoes R. A population dynamics analysis of the interaction between adaptive regulatory T cells and antigen presenting cells. *PLoS One* (2008) **3**:e2306. doi:10.1371/journal.pone.0002306

3. Vélez de Mendizábal N, Carneiro J, Solé R V, Goñi J, Bragard J, Martinez-Forero I, Martinez-Pasamar S, Sepulcre J, Torrealdea J, Bagnato F, et al. Modeling the effector - regulatory T cell cross-regulation reveals the intrinsic character of relapses in Multiple Sclerosis. *BMC Syst Biol* (2011) **5**:114. doi:10.1186/1752-0509-5-114

4. Chen W, Jin W, Hardegen N, Lei K, Li L, Marinos N, McGrady G, Wahl SM. Conversion of Peripheral $CD4^{+}$ $CD25^{-}$ Naive T Cells to $CD4^{+}$ $CD25^{+}$ Regulatory T Cells by TGF-β Induction of Transcription Factor *Foxp3*. *J Exp Med* (2003) **198**:1875–1886. doi:10.1084/jem.20030152

5. Zilman A, Ganusov V V., Perelson AS. Stochastic Models of Lymphocyte Proliferation and Death. *PLoS One* (2010) **5**:e12775. doi:10.1371/journal.pone.0012775

6. De Boer RJ, Ganusov V V., Milutinović D, Hodgkin PD, Perelson AS. Estimating Lymphocyte Division and Death Rates from CFSE Data. *Bull Math Biol* (2006) **68**:1011–1031. doi:10.1007/s11538-006-9094-8
